# Geospatial Visualization of Dialysis Accessibility in Shiraz: A Nonanalytical Geographic Information System Approach

**DOI:** 10.1002/hsr2.71137

**Published:** 2025-08-07

**Authors:** Peyman Ghahramani, Behzad Kiani, Narges Norouzkhani, Atefeh Khoshkangin, Azam Kheirdoust, Mohammad Reza Mazaheri Habibi

**Affiliations:** ^1^ Department of Health Information Technology Varastegan Institute for Medical Sciences Mashhad Iran; ^2^ Department of Medical Informatics, School of Medicine Mashhad University of Medical Sciences Mashhad Iran; ^3^ Department of Medical Informatics, Faculty of Medicine Mashhad University of Medical Sciences Mashhad Iran

**Keywords:** dialysis, Geographic Information Systems (GIS), healthcare accessibility, hemodialysis, spatial inequality

## Abstract

**Background and Aims:**

Chronic kidney disease (CKD) poses a substantial global health challenge, contributing to increased morbidity and mortality, diminished quality of life, and escalating healthcare expenditures. Despite advancements in nephrology and dialysis technologies, disparities in hemodialysis (HD) accessibility remain prevalent, leading to suboptimal patient outcomes and increased healthcare burdens. Geographic Information Systems (GIS) facilitate the spatial analysis of healthcare service distribution, identifying inequities in access. This study employs GIS to evaluate the spatial distribution of dialysis facilities in Shiraz, highlight underserved areas, and assess geographic barriers impacting patient access.

**Methods:**

A GIS‐based spatial analysis was performed utilizing deidentified patient demographics, dialysis facility locations, transportation network data, and urban zoning characteristics to assess accessibility and service distribution. Sophisticated geospatial methodologies, including Network Analysis and Kernel Density Estimation (KDE), were employed to model travel time variations and evaluate spatial equity in‐service distribution. The study adhered to SAMPL and CONSORT reporting guidelines.

**Results:**

Among 605 patients (mean age: 60.9 ± 15.4 years; 64.6% male), substantial spatial disparities in dialysis service accessibility were identified, with notable variations in travel burden and facility distribution. Patients from socioeconomically disadvantaged neighborhoods experienced prolonged travel times and increased transportation expenses, further intensifying healthcare access disparities. GIS‐based spatial modeling identified priority zones for service expansion, proposing targeted interventions to optimize resource allocation.

**Conclusion:**

Socioeconomic inequities substantially impact dialysis accessibility, concentrating patients in cost‐effective residential areas with heightened travel burdens and delayed treatment initiation. GIS‐driven spatial planning provides a data‐driven framework for equitable dialysis resource allocation, facilitating evidence‐based healthcare policy decisions.

**Abbreviations**
CKD: chronic kidney disease
ESRD: end stage renal disease
GIS: Geographic Information Systems
HD: hemodialysis
PD: peritoneal dialysis

## 1 Introduction

Chronic kidney disease (CKD) is a progressive condition that not only affects physical health but also impairs psychological and social well‐being [1]. Patients often remain asymptomatic during the early stages, exhibiting complications associated with kidney dysfunction only in its advanced phases [2, 3]. These complications are linked to increased mortality rates and heightened cardiovascular risks [4, 5, 6, 7, 8, 9], alongside a substantial decline in the quality of life [4, 5].

Hemodialysis (HD) is a vital renal replacement therapy that utilizes extracorporeal blood circulation to manage *uremia*‐related abnormalities, including fluid overload, azotemia, electrolyte imbalances, and acid–base disturbances characteristic of *uremic syndrome*. HD is primarily employed for the treatment of acute and chronic kidney failure that is unresponsive to conventional medical therapies [10].

Considering that patients typically undergo HD three times per week, each session lasting 3–5 h, even moderate travel times compound to a significant weekly time burden, exacerbating treatment fatigue and potentially compromising adherence.

The frequency and duration of these treatments are critical to ensuring optimal toxin and fluid removal from the body. Regular and adequate dialysis prevents the accumulation of harmful substances, thereby improving patient outcomes. However, the demanding nature of frequent sessions makes geographical proximity to dialysis centers a crucial factor. Long travel times lead to patient fatigue and increase the likelihood of missed appointments, directly compromising treatment efficacy.

The economic burden of HD further complicates accessibility. As one of the most expensive therapeutic interventions for patients with end‐stage renal disease (ESRD), HD requires significant financial resources [11]. The financial structure of dialysis centers plays a pivotal role in determining patient access. While some centers are publicly funded through governmental healthcare programs, others operate privately. This division influences dialysis services' availability, affordability, and overall quality. In many healthcare systems, governmental bodies or specialized health authorities are responsible for strategically planning and placing dialysis centers. In Shiraz, dialysis facility planning is overseen by the Ministry of Health and Medical Education (MOHME) in collaboration with the Shiraz University of Medical Sciences, which assesses population needs and allocates resources accordingly. These agencies assess patient density, geographic distribution, and available resources to optimize facility locations and capacities.

Patients with ESRD often choose between *peritoneal dialysis* (PD) and HD, depending on various factors such as medical indications, patient preferences, and logistical considerations. Geographic proximity to HD centers significantly impacts this decision‐making process, as longer distances can deter patients from selecting HD as their primary treatment modality [12].

Geographical and temporal elements are critical determinants of dialysis accessibility. The spatial gap between a patient's residence and the nearest dialysis center can pose significant barriers, particularly in regions lacking efficient public transportation or where urban sprawl complicates travel. The necessity of attending dialysis sessions 2–3 times/week, each lasting several hours, imposes a considerable strain on patients, especially when compounded by long travel distances. In cities like Shiraz, unique geographical challenges—such as uneven urban expansion and insufficient public transportation infrastructure in certain districts—hinder regular dialysis service access. Preliminary spatial reviews indicate that southern and western districts of Shiraz experience disproportionately lower dialysis accessibility compared to central areas. This underscores the importance of employing Geographic Information Systems (GIS) to identify underserved areas and guide equitable resource allocation systematically.

GIS is a robust analytical tool integrating diverse spatial, temporal, and descriptive data sets within a unified framework [13, 14, 15]. This technology has been extensively employed in healthcare for research, education, planning, and the monitoring of public health programs. It plays a vital role in understanding disease patterns, managing healthcare resources, and addressing disparities in access to medical services [16, 17, 18, 19]. In dialysis care, GIS enables a nuanced understanding of how factors such as residential location, transportation networks, and the spatial distribution of dialysis centers influence patient accessibility. Its applications range from creating simple epidemiological maps to conducting advanced spatial analyses that inform policy decisions and optimize healthcare delivery [20].

Optimizing dialysis center placement through GIS‐based analysis can significantly reduce patient travel times, enhance adherence to treatment regimens, and improve health outcomes. Strategically located centers alleviate the physical and emotional burden on patients and contribute to the efficiency and sustainability of healthcare systems.

This study aims to evaluate the spatial distribution of dialysis centers in Shiraz using GIS and propose actionable recommendations for improving patient access and promoting *health equity* through evidence‐based planning and resource allocation.

## 2 Methods

This GIS‐based cross‐sectional study aimed to evaluate the spatial accessibility and distribution of dialysis centers in Shiraz, providing evidence‐based recommendations to improve equitable access for patients with CKD.

This study was conducted with the support and approval of the Varastegan Institute for Medical Sciences, in collaboration with Mashhad and Shiraz University of Medical Sciences, and with permission from the Ethics Committee (IR.MUMS.REC.1399.596). Informed consent was obtained from all participants involved in the study, ensuring adherence to ethical standards. All study procedures followed the Declaration of Helsinki and other relevant ethical guidelines. For participants under 16, consent was secured from a parent or legal guardian. Written consent was documented for all participants. The anonymity and confidentiality of participant data were strictly maintained throughout the research process.

The data set used in this study was obtained from the *Kidney Patients Association of Fars Province* in the form of an Excel file. This data set contained demographic data, including first and last names, genders, ages, residential addresses, contact information, type of insurance, cause of kidney failure, and the dialysis center to which each patient was referred.

The data set included 605 participants, with 391 males (64.6%) and 214 females (35.4%). The average age of the participants was 60.86 ± 15.43 years. The distribution of the causes of kidney failure was as follows: 180 participants (29.8%) had high blood pressure, 93 participants (15.4%) had both high blood pressure and diabetes, and 87 participants (14.4%) had diabetes alone. Less common causes included kidney stones (3.1%), polycystic kidney disease (2.8%), urological problems (2.1%), nephrotic syndrome (1.5%), glomerulonephritis (1%), congenital issues (1%), and kidney failure caused by COVID‐19 (0.3%). Additionally, 28.3% of participants had other unspecified conditions or unknown causes.

Occupational data revealed that 212 participants (35%) were self‐employed, 155 (25.6%) were housewives, and 96 (15.9%) were retired. The remainder included unemployed individuals (6.1%), employees (4%), workers (1.5%), students (1%), farmers (0.8%), and one participant (0.2%) listed as a student. Unknown occupations accounted for 9.9% of the data set.

The data set was thoroughly reviewed before analysis to ensure data accuracy and completeness. The residential addresses of patients were geocoded using the *Google Maps API*, transforming them into geospatial data points. This process enabled mapping the locations of patients on a geographic scale, allowing for spatial analysis of patient distribution across Shiraz. In parallel, the addresses of all dialysis centers in Shiraz were also geocoded and stored in a separate GIS layer explicitly designated for dialysis centers.

GIS tools integrated patients' geospatial data with the dialysis centers' mapped locations. Accessibility analysis measured travel distance and estimated travel time between each patient's residence and the nearest dialysis center, combined with spatial density mapping of dialysis services. This integration facilitated comprehensive spatial analyses to evaluate patient access to dialysis services. The GIS analysis enabled the calculation of travel distances between patient residences and dialysis centers, the identification of underserved regions, and the assessment of spatial disparities in access to dialysis care.

Specifically, the analysis focused on determining the proximity of patients to dialysis centers, identifying areas with high patient density but limited access to dialysis services, and evaluating geographical gaps in service availability to inform potential locations for new dialysis centers.

An analysis of the GIS data highlighted a significant gap in the use of this technology within healthcare systems, particularly in determining optimal locations for essential services such as hospitals, dialysis centers, urban health centers, and rural clinics. Implementing GIS could address this gap, ensuring equitable and efficient distribution of healthcare facilities.

For statistical analysis, descriptive statistics, including mean (*μ*), median, and standard deviation (*σ*), were calculated for demographic and clinical variables. Inferential statistical tests were also conducted to explore relationships between patient characteristics and their proximity to dialysis centers. Chi‐square (*χ* ^2^) tests were used to analyze categorical variables, while correlation analyses were employed to examine associations between continuous variables. Two‐sided statistical tests were performed with a significance level set at *p* < 0.05. *p* values were reported following standard conventions: for *p* < 0.001, values were reported as *p* < 0.001; for *p* between 0.001 and 0.01, values were reported to the nearest thousandth; and for *p* ≥ 0.01, values were reported to the nearest hundredth.

All statistical analyses were conducted using SPSS version 26 (IBM Corp., Armonk, NY, USA). The statistical procedures followed the guidelines outlined in [21] and adhered to the *SAMPL* reporting standards for clinical research.

Data selection was based on the availability of comprehensive and reliable patient information, including geospatial data. Only patients currently undergoing dialysis were included in the analysis. Missing data were handled using listwise deletion, ensuring that only complete cases were analyzed for each statistical test.

**Figure 1:**
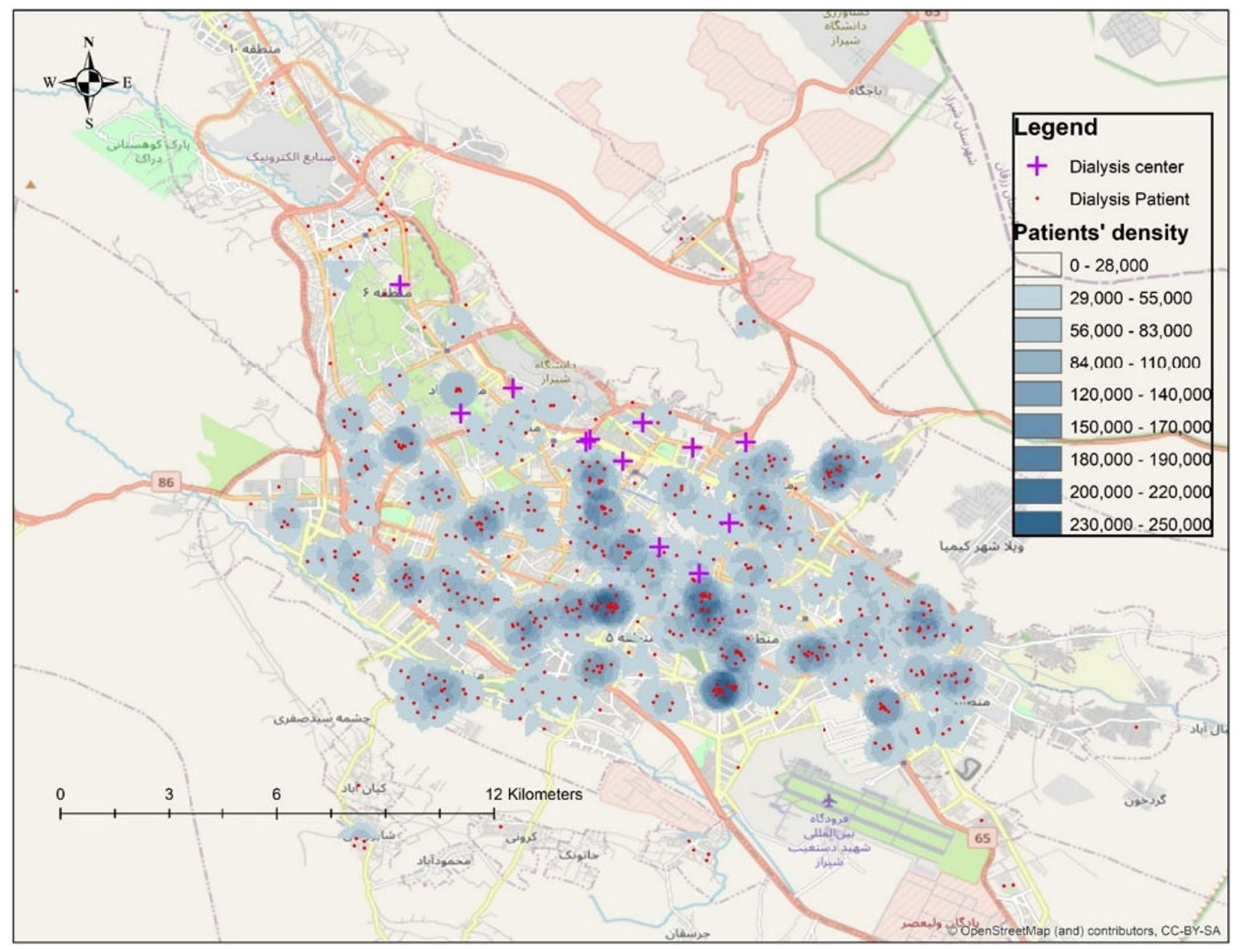
Distribution of patients and distribution of dialysis centers.

The findings were visualized using GIS‐generated maps, which depicted the spatial distribution of dialysis centers relative to patient residences. These maps highlighted areas with limited access to dialysis services and were used to recommend strategic locations for establishing new dialysis centers. Figure 1 illustrates the spatial distribution of patients and dialysis centers in Shiraz, emphasizing regions with high patient density but low service availability.

Combining geospatial and statistical analyses, this comprehensive approach provides valuable insights into the accessibility of dialysis services in Shiraz and offers actionable recommendations for optimizing the placement of future dialysis centers. The generated maps and spatial analyses are intended to guide health policymakers and planners in Shiraz in identifying priority zones for future dialysis center development, ensuring a more equitable distribution of services.

## 3 Results

### 3.1 Demographic and Clinical Characteristics

A total of 605 participants were included in this study, comprising 391 males (64.6%) and 214 females (35.4%). The average age of participants was 60.86 ± 15.43 years.

The leading cause of kidney failure was hypertension (180 participants; 29.8%), followed by combined hypertension and diabetes (93 participants; 15.4%) and diabetes alone (87 participants; 14.4%). Other contributing causes included kidney stones (19 participants; 3.1%), polycystic kidney disease (17 participants; 2.8%), urological problems (13 participants; 2.1%), nephrotic syndrome (9 participants; 1.5%), glomerulonephritis (6 participants; 1%), congenital anomalies (6 participants; 1%), and COVID‐19‐related kidney failure (2 participants; 0.3%). Additionally, 171 participants (28.3%) had other or unspecified causes.

Regarding occupational status, 212 participants (35%) were self‐employed, 155 (25.6%) were housewives, and 96 (15.9%) were retired. Smaller proportions were unemployed (6.1%), employed (4%), workers (1.5%), students (1%), and farmers (0.8%), while occupational data were unavailable for 60 participants (9.9%). Table 1 presents a detailed summary of demographic and clinical characteristics.

**Table 1:** Demographic and clinical characteristics of patients.

Variable	*n* (%)
Total patients	605 (100%)
Gender	
Male	391 (64.6%)
Female	214 (35.4%)
Mean age (±SD)	60.86 ± 15.43 years
Cause of kidney failure	
Hypertension	180 (29.8%)
Hypertension + diabetes	93 (15.4%)
Diabetes	87 (14.4%)
Kidney stones	19 (3.1%)
Polycystic kidney disease	17 (2.8%)
Urological problems	13 (2.1%)
Nephrotic syndrome	9 (1.5%)
Glomerulonephritis	6 (1%)
Congenital issues	6 (1%)
COVID‐19 related	2 (0.3%)
Unknown/other	171 (28.3%)
Occupational status	
Self‐employed	212 (35%)
Housewives	155 (25.6%)
Retired	96 (15.9%)
Unemployed	37 (6.1%)
Employees	24 (4%)
Workers	9 (1.5%)
Students	6 (1%)
Farmers	5 (0.8%)
Unknown	60 (9.9%)

### 3.2 GIS‐Based Spatial Analysis of Dialysis Accessibility

To evaluate spatial accessibility, geocoded residential addresses of patients and dialysis center locations were analyzed using GIS. Figure 1 illustrates the geographic distribution of dialysis patients and centers across Shiraz.

The spatial analysis revealed a concentration of dialysis patients in central and southern districts, with relatively lower densities in peripheral areas. Dialysis centers were also predominantly clustered in central areas, resulting in noticeable service gaps in outer districts, particularly in southwestern and southeastern zones of Shiraz.

Kernel Density Estimation (KDE) was employed to identify high‐density patient areas with limited access to dialysis centers. The results indicate that underserved areas in Shiraz, particularly peripheral districts, should be prioritized for future dialysis center development to enhance accessibility and reduce travel burden.

### 3.3 Statistical Analysis of Patient Characteristics and Access

Descriptive statistics were calculated for demographic and clinical variables (Table 1). *χ* ^2^ analysis showed no significant association between gender and the cause of kidney failure (*χ* ^2^ = 10.73, *p* = 0.47), indicating that the distribution of kidney failure causes was similar between male and female patients.

Additionally, no significant difference in mean age was found between male and female participants (*p* > 0.05). Correlation analysis revealed no meaningful relationship between age and the number of comorbid conditions (*r* = 0.05), suggesting that older patients were not significantly more likely to have multiple contributing factors to kidney failure.

### 3.4 Geographic Inequities and Resource Allocation

These findings underscore the need for evidence‐based spatial planning to achieve a more equitable distribution of dialysis services across Shiraz. By utilizing GIS to guide healthcare policy decisions, health authorities can identify high‐need areas and strategically plan the placement of future dialysis centers to optimize service accessibility. To enhance transparency and reproducibility, the complete data set and metadata are publicly available in the Harvard Dataverse Repository (https://doi.org/10.7910/DVN/K7XWON23), ensuring that researchers and policymakers can access and build upon this study.

## 4 Discussion

Effective management of healthcare resources is vital in improving access to essential services and reducing healthcare costs [22]. This is particularly critical for facilities like dialysis centers, which serve vulnerable populations requiring regular care. In developing countries such as Iran, healthcare facility locations are often selected without systematic spatial analysis, leading to inequitable access and increased patient burden. Shiraz, as one of Iran's major cities, exemplifies this challenge, especially in the context of dialysis services for ESRD patients.

Our study applied GIS‐based spatial analysis to address this gap, highlighting the need for evidence‐based, data‐driven approaches in the placement of dialysis centers.

The spatial visualization (Figure 1) revealed the clustering of dialysis patients in central and southern Shiraz, with clear service gaps in peripheral districts, especially in the northwest and southeast. KDE further identified priority areas where high patient density coincided with low service coverage, underscoring the need for new facilities in these underserved zones.

These findings align with previous studies. For example, Kiani and colleagues applied GIS to evaluate dialysis access in northeastern Iran, identifying similar urban–rural disparities [23]. Matsumoto and colleagues also highlighted the importance of combining travel time and facility capacity in dialysis access assessments [24], a principle echoed in our work, particularly for peripheral districts facing prolonged commutes.

Furthermore, Yang and colleagues emphasized that advanced spatial methods, such as the Two‐Step Floating Catchment Area (2SFCA), can provide more nuanced insights into healthcare access [25]. While our study applied simpler density‐based mapping, the same inequity patterns were revealed, reinforcing the validity of our findings.

Our results (Table 1) also showed that socioeconomically disadvantaged patients—including unemployed individuals and those with lower incomes—are disproportionately concentrated in areas with poorer dialysis access. This aligns with Kiani et al. [23] and multiple UK‐based studies [26, 27, 28, 29], which demonstrated that socioeconomic deprivation exacerbates barriers to healthcare access.

Elderly patients, who often have limited mobility, were also overrepresented in these underserved districts, compounding the access challenges. This dual burden of spatial and socioeconomic inequity calls for integrated policies addressing geographic gaps and financial barriers to care.

Prolonged travel distances, especially for patients in peripheral districts, significantly contribute to treatment fatigue and missed appointments—both directly impact clinical outcomes. This is consistent with findings from Stephens and colleagues, who reported that rural dialysis patients in the USA faced longer travel times and poorer outcomes than their urban counterparts [30].

Our analysis reinforces this concern, demonstrating that patients in outer districts travel more than double the distance compared to centrally located patients, exacerbating physical, financial, and emotional stress.

GIS is not merely a visualization tool; it provides actionable insights for policymakers. By integrating patient distribution data, facility locations, and travel patterns, GIS enables health authorities to identify optimal sites for future dialysis centers, ensuring alignment between patient demand and service capacity.

This evidence‐based approach is particularly critical in dynamic urban environments like Shiraz, where rapid population growth and urban sprawl continuously reshape healthcare needs. Our study advocates institutionalizing GIS‐based spatial analysis within health planning frameworks to enhance equity and efficiency in healthcare resource allocation.

Beyond spatial planning, addressing socioeconomic disparities is essential. This requires incorporating income, education, and employment data into health planning processes, ensuring that new facilities are not only geographically accessible but also financially and culturally accessible to all population groups.

Technological solutions, including telemedicine and mobile health (mHealth) applications, can further support equitable access. These technologies enable remote monitoring, appointment scheduling, and real‐time information on facility availability, which is particularly beneficial for patients in remote areas. Such innovations proved invaluable during the COVID‐19 pandemic [31, 32, 33, 34, 35], and their integration into routine dialysis care could alleviate some travel burdens identified in this study.

The GIS‐based findings of this study offer direct, actionable recommendations for health policymakers in Shiraz: prioritize the establishment of new dialysis centers in high‐need peripheral areas identified through KDE analysis. Enhance patient transportation services, such as subsidized transit or mobile dialysis units, to alleviate travel burdens. Integrate GIS into routine health planning workflows to monitor healthcare access gaps dynamically. Combine spatial analysis with socioeconomic profiling to ensure both geographic and financial equity.

Accurate equity in healthcare access requires a multidimensional approach that combines spatial analysis with real‐time demographic, socioeconomic, and health system data. Future research should develop composite access indices that account for travel time, financial barriers, facility capacity, and patient preferences, providing policymakers with holistic decision‐making tools.

Our study highlights the critical role of GIS in identifying and addressing spatial disparities in dialysis access in Shiraz. By combining spatial visualization with density‐based analysis, we demonstrated clear geographic inequities exacerbated by underlying socioeconomic challenges. Integrating GIS into health policy frameworks alongside socioeconomic and technological interventions offers a robust path toward achieving equitable, efficient, and patient‐centered healthcare delivery.

### 4.1 Limitations

This study has several limitations. First, the analysis was limited to patients residing within Shiraz City, excluding those referred from neighboring cities or provinces. Patients who had changed addresses multiple times within 6 months were also excluded to ensure data consistency and spatial accuracy.

Second, the study did not incorporate data on the operational capacity, staffing levels, or service quality of existing dialysis centers—factors that could significantly influence patient preferences and accessibility. Including such variables in future research would provide a more comprehensive understanding of dialysis service accessibility.

Thirdly, although the study highlighted socioeconomic inequities using demographic data, socioeconomic variables such as household income, educational attainment, and transportation affordability were not directly incorporated into the GIS analysis. This omission limits the ability to fully capture nonspatial barriers to care, including financial and informational obstacles that influence healthcare access.

Additionally, the methodological framework employed in this study was primarily descriptive and did not incorporate advanced spatial analytical techniques such as the 2SFCA method, Moran's *I* spatial autocorrelation, hotspot analysis (e.g., Getis‐Ord Gi*), or multicriteria decision analysis.

The exclusion of these techniques limits the analytical depth of the study and constrains its utility as a robust decision‐support tool.

This limitation was mainly due to data unavailability and the exploratory nature of the research. Future investigations should consider adopting such techniques to enable more comprehensive and inferential spatial assessments of service accessibility.

Furthermore, travel distances were calculated using GIS tools based on the shortest road network routes; however, real‐time traffic patterns, road congestion, and public transportation schedules were not considered. These factors may significantly impact actual travel times, particularly during peak hours. Future studies should consider integrating dynamic traffic data to improve the accuracy of travel time estimations.

## 5 Conclusion

Strategic management of healthcare resources is essential for enhancing accessibility and reducing healthcare costs, particularly in expanding urban centers like Shiraz. Our GIS‐based spatial analysis identified substantial geographic disparities in access to dialysis services, demonstrating the urgent need for new dialysis centers in underserved areas.

The results further emphasize how socioeconomic dynamics—such as migrating lower‐income populations to peripheral neighborhoods with lower living costs—have exacerbated these spatial disparities. This dual burden of geographic and financial barriers highlights the need for integrated spatial and socioeconomic planning approaches to ensure equitable access to essential healthcare services.

Spatial analyses, like the one conducted in this study, provide health authorities and policymakers with objective, evidence‐based insights to optimize the placement of new dialysis centers and improve service equity. By institutionalizing GIS‐based spatial analysis within routine healthcare planning, cities like Shiraz can develop more responsive, equitable, and efficient healthcare delivery systems.

To further contribute to the evidence base and support future research and planning efforts, we intend to publish the full data set and spatial layers, enabling researchers and health planners to build upon our findings and develop more advanced models for healthcare accessibility analysis in Shiraz and comparable urban settings.

## Author Contributions

**Peyman Ghahramani:** investigation, writing – original draft, software. **Behzad Kiani:** investigation, software, formal analysis, validation, methodology. **Narges Norouzkhani:** writing – original draft, investigation, writing – review and editing, software, methodology. **Atefeh Khoshkangin:** writing – original draft, writing – review and editing, formal analysis, methodology. **Azam Kheirdoust:** investigation, writing – original draft, methodology, formal analysis. **Mohammad Reza Mazaheri Habibi:** conceptualization, writing – review and editing, methodology, project administration, formal analysis. All authors read, revised, and approved the final manuscript.

## Ethics Statement

This study was approved by the Ethical Committee of Mashhad University of Medical Sciences (Approval number: IR.MUMS.REC.1399.596). All methods followed relevant guidelines and regulations (including the Declaration of Helsinki).

## Consent

Informed consent was obtained from all participants before being included in the study. For participants under 16, consent was obtained from a parent or legal guardian. All consent forms were documented in writing and securely stored.

## Conflicts of Interest

The authors declare no conflicts of interest.

## Transparency Statement

The lead author Mohammad Reza Mazaheri Habibi affirms that this manuscript is an honest, accurate, and transparent account of the study being reported; that no important aspects of the study have been omitted; and that any discrepancies from the study as planned (and, if relevant, registered) have been explained.

## Supporting information

Supporting File.
**Table 2:** Overview of Data Files/Data Sets.

## Data Availability

The data set supporting the conclusions of this article is publicly available in the Harvard Dataverse repository under the following link: https://doi.org/10.7910/DVN/K7XWON [22]. This data set includes anonymized demographic data, spatial coordinates, and other relevant attributes used in the GIS analysis. For further details, please refer to Table S2 and the reference list. M.R.M.H. had full access to all data and was responsible for the data analysis's integrity, completeness, and accuracy.
