# Supplementary material for: Geospatial Visualization of Dialysis Accessibility in Shiraz: A Nonanalytical Geographic Information System Approach
Source: Health Sci Rep. 2025 Aug 7;8(8):e71137. doi: 10.1002/hsr2.71137 (PMC12331525; doi:10.1002/hsr2.71137)
Supplement: Supplementary file 1 — Supporting File. Table 2: Overview of Data Files/Data Sets. [file HSR2-8-e71137-s001.docx]

**Supplementary File**

**Table 2: Overview of Data Files/Data Sets**

| **Label** | **Name of Data File/Data Set** | **File Types** | **Data Repository and Identifier** | **Description** |
| --- | --- | --- | --- | --- |
| Data file 1 | Patients_Data | Excel file (*.xlsx) | Harvard Dataverse <https://doi.org/10.7910/DVN/K7XWON23> | Contains demographic and clinical data of *dialysis* patients. |
| Data file 2 | Help file | Excel file (*.xlsx) | Harvard Dataverse <https://doi.org/10.7910/DVN/K7XWON23> | Provides metadata and codebook for data interpretation. |
